# Supplementary material for: The Running Injury Continuum: A qualitative examination of recreational runners’ description and management of injury
Source: PLoS One. 2023 Oct 4;18(10):e0292369. doi: 10.1371/journal.pone.0292369 (PMC10550191; doi:10.1371/journal.pone.0292369)
Supplement: S1 Table — (DOCX) [file pone.0292369.s001.docx]

**S1 Table. Focus group schedule, introduction and aims.**

| **Domain** | **Sample dialogue** |
| --- | --- |
| Introduction & aims of study | Hi everyone. Thank you for coming and for being involved in this study. I am conducting some research on runners’ description and management of injury, and the aim of this study is to gather your thoughts on running-related injuries. Please go into as much detail as you can, ask each other questions, and agree or disagree on any points raised, but please respect everyone’s opinion. If you have any questions, please ask at any point. |
| Sample questions | How would you define injury? |
|  | How would you describe injury? |
|  | Based on your descriptions of injury and the terms you have used, could you elaborate on these on the whiteboard? |
|  | How would you manage injuries? |
