# Supplementary material for: The Running Injury Continuum: A qualitative examination of recreational runners’ description and management of injury
Source: PLoS One. 2023 Oct 4;18(10):e0292369. doi: 10.1371/journal.pone.0292369 (PMC10550191; doi:10.1371/journal.pone.0292369)
Supplement: S1 Fig — AL: Aisling Lacey, SOK: Sinéad O’Keeffe, EW: Enda Whyte, KM: Kieran Moran, SOC: Siobhán O’Connor, AB: Aoife Burke. (DOCX) [file pone.0292369.s003.docx]

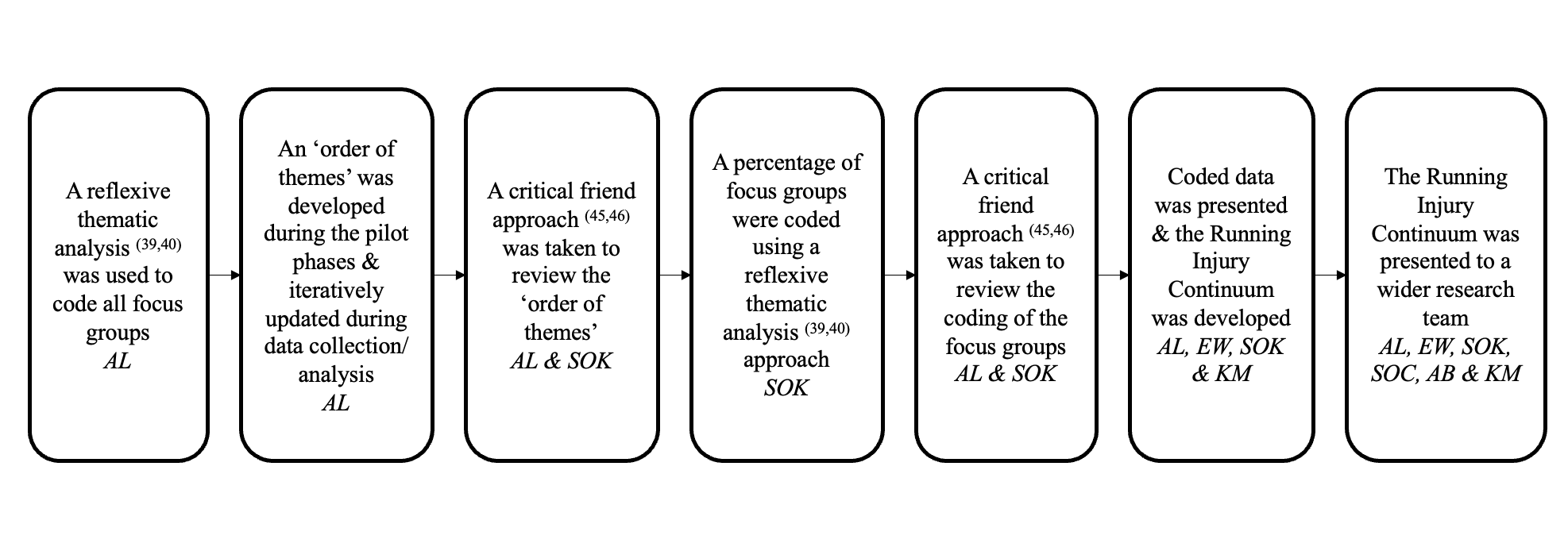


**S1 Fig. Data analysis and the Running Injury Continuum development process.** AL: Aisling Lacey, SOK: Sinéad O’Keeffe, EW: Enda Whyte, KM: Kieran Moran, SOC: Siobhán O’Connor, AB: Aoife Burke.
